# The Dynamic Transcription Profiles of Proliferating Bovine Ovarian Granulosa When Exposed to Increased Levels of β-Hydroxybutyric Acid

**DOI:** 10.3389/fvets.2022.915956

**Published:** 2022-08-05

**Authors:** Jianfei Gong, Shanjiang Zhao, Nuo Heng, Yi Wang, Zhihui Hu, Huan Wang, Huabin Zhu

**Affiliations:** State Key Laboratory of Animal Nutrition, Key Laboratory of Animal Genetics, Breeding and Reproduction of Ministry of Agriculture and Rural Affairs, Institute of Animal Science, Chinese Academy of Agricultural Sciences, Beijing, China

**Keywords:** cow, ketosis, β-hydroxybutyric acid, granulosa cells, proliferation, RNA-seq, trend analysis

## Abstract

Ketosis is common in high-yield dairy cows. It is a condition that is characterized by the accumulation of serum β-hydroxybutyric acid (BHBA). Both subclinical ketosis and clinical ketosis can compromise the reproductive performance and cause long-lasting negative effects on reproductive efficiency by affecting the proliferation of follicular and granulosa cells. However, the regulatory mechanisms involved in the development of follicular cells and granulosa cells in cows experiencing subclinical ketosis and clinical ketosis remain largely unknown. To investigate the effect of a ketosis-triggered increase in BHBA on bovine follicular granulosa cell development, we detected a significant reduction in the proliferation of granulosa cells (*P* < 0.05) in the BHBA-1.2 m*M* and BHBA-2.4 m*M* groups and a significant increase in the number of granulosa cells in the G1 phase of the cell cycle (*P* < 0.05). RNA-seq and trend analysis were used to identify differentially expressed genes by comparing three clusters: low-concentration response to 1.2 m*M* BHBA, high-concentration response to 2.4 m*M* BHBA, and the similar trend (up or down) response following BHBA concentration increased. GO and KEGG enrichment analyses were performed separately for each cluster. Analysis showed that two novel down-regulated genes (*G0S2* and *S100A6*), which are associated with cell proliferation and cycle progression, were enriched in the low-concentration response to 1.2 m*M* BHBA. Another differentially expressed gene (*PARP*), which plays a role in the apoptotic pathway, was enriched in the high-concentration response to 2.4 m*M* BHBA. We also found that *CYP27B1* and *CYP17A1*, which are associated with Ca^2+^ homeostasis and estrogen synthesis, were enriched in a similar trend response. In conclusion, we describe the dynamic transcription profiles of granulosa cells under different levels of β-hydroxybutyric stress and report key regulators that may underlie the detrimental effects on the development of follicles and granulosa cells, thus representing potential therapeutic targets to improve fertility in dairy cows with subclinical ketosis or clinical ketosis.

## Introduction

After calving, all cows (especially high-yielding cows) experience a negative energy balance (NEB). This is due to an insufficiency in the dry matter intake (DMI) and high levels of lactation. If cows cannot adapt to NEB in a timely manner, they are prone to metabolic disorders and produce excess ketone bodies (KBs), which may ultimately develop ketosis (1–4). Ketosis is characterized by an increase of KBs in the blood, with β-hydroxybutyric acid (BHBA) being approximately 70% of the KBs (5). Due to its relative stability in KBs, BHBA is commonly used to distinguish between healthy and ketotic cows. If the concentration of BHBA in the serum is between 1.2 mmol·L^−1^ (m*M*) and 1.4 mmol·L^−1^ (m*M*), and there are no clinical signs (6–8), then a cow is diagnosed with subclinical ketosis (SCK). When the BHBA concentration exceeds 1.4 mM and there are obvious clinical signs (such as standing behavior) (8), then a cow is diagnosed with clinical ketosis (CK). Research has also shown that ketosis not only reduces the production of milk, it can also reduce the reproductive performance of cows, thus leading to serious economic losses. This is particularly the case for SCK, which has been referred to as a “silent profit robber” because it has no obvious clinical signs (9, 10).

Ketosis can reduce physical activity around the time of estrus in dairy cows and prolong the interval from calving to the first service (10, 11). This may be related to the high levels of BHBA which can damage granulosa cells (GCs) and inhibit follicular development, thus leading to a disrupted estrus cycle and long-term effects on reproductive efficiency (12–14). It is well known that ovarian GCs are vital to estrogen synthesis, follicular growth, oocyte development, ovulation, and luteinization (15, 16). Thus, maintaining a balance between cell proliferation and apoptosis is essential for GCs as this also determines the fate of follicles. Existing data suggest that an abnormal increase in BHBA may lead to intracellular oxidative stress and ultimately induce apoptosis in somatic cell models, such as bovine abomasum smooth muscle cells and bovine liver cells (17–19). However, metabolic stress, involving elevated concentrations of BHBA, has been shown to cause apoptosis in ovine GCs, thus inhibiting estrogen synthesis and negatively affecting fertility (20). Studies involving direct stimulation of GCs through increased BHBA to explore the mechanisms of their effects on fertility are still scarce. In this study, we validated the effects of different concentrations of BHBA on the proliferation and cell cycle of GCs *in vitro*. We also explored the dynamic transcriptional profile of GCs in response to the increasing concentration of BHBA stimulation. We intended to provide new insights into the molecular mechanisms responsible for the reduced reproductive performance of dairy cows with SCK and CK induced by increases in BHBA.

## Materials and Methods

### Ethics Statement

All the animal procedures used in this study were approved by the Animal Care and Use Committee of the Institute of Animal Sciences of the Chinese Academy of Agricultural Sciences.

### Isolation, Culture, and Characterization of GCs

Ovaries from dairy cows were collected from an abattoir and washed with preheated 0.9 % NaCl solution (containing 100 U/ml of penicillin and 0.1 mg/ml of streptomycin) at 37°C. Peripheral ovarian tissue was removed with a sterile surgical scissor and then rinsed in 75% alcohol. Next, the ovaries were washed 4–5 times with NaCl (100 U/ml of penicillin and 0.1 mg/ml of streptomycin). Finally, the ovaries were washed with preheated phosphate-buffered saline (PBS) (C10010500BT, Gibco). Healthy follicles, 2–6 mm in diameter, were extracted using a syringe on an ultra-clean table and transferred to a 15 ml centrifuge tube (430790, Corning). The collected follicular fluid was then filtered into a new 15 ml centrifuge tube and washed three times with Dulbecco's phosphate-buffered saline (DPBS) (14190-144, Gibco). After centrifugation at 1,500 rpm for 5 min, a precipitate was left; GCs were then re-suspended in preheated Dulbecco's Modified Eagle Medium (DMEM)/F12 (11330032, Gibco) complete medium (with 10% fetal bovine serum (FBS, 10099141C, Gibco) and 1% penicillin-streptomycin) at 37°C. Subsequently, the GCs were cultured at 37°C for 24 h under 5% CO_2_, and the cell medium was renewed every 24 h.

In this study, we used immunofluorescence to detect follicle-stimulating hormone receptors (FSHRs) for characterizing the GCs (21). The GCs were cultured in a confocal dish for 48 h and then washed three times with DPBS. At room temperature, GCs were fixed with 4% paraformaldehyde for 1 h and then permeabilized with 0.5% Triton X-100 for 40 min. PBS with 1% bovine serum albumin (BSA) was used to block the GCs overnight at 4°C; the GCs were then incubated overnight with specific antibodies against FSHR (1:500, 22665-1-AP, Proteintech) at 4°C. The following morning, the GCs were incubated with fluorescein-conjugated Goat Anti-Rabbit IgG (1:500, P03S06S, Gene-Protein Link). The nuclei of the GCs were incubated with DAPI (C1006, Beyotime) for 5 min. Cells were then observed and representative images were captured using a Leica SP8 fluorescence inverted microscope. Representative results are shown in Supplementary Figure 1.

### BHBA Incubation

The concentration of BHBA in the control group was 0 m*M*, and in the treatment groups, 1.2 m*M* and 2.4 m*M* respectively. First, the GCs were cultured in 6-well plates for 48 h and then starved in an FBS-free medium for 12 h. Next, fresh DMEM/F12 medium (with 2% FBS) containing 1.2 m*M* and 2.4 m*M* BHBA (H6501-5G, Sigma) was added, and the cells were cultured for 12 h. GCs cultured in DMEM/F12 medium (with 2% FBS, without BHBA) were used as a control. Each treatment concentration of BHBA was replicated three times. Finally, GCs were collected with 0.25% trypsin (25200056, Gibco) and stored at −80°C to await RNA extraction.

### Cell Proliferation Detection

The BeyoClick™ EdU-488 Cell Proliferation Assay Kit (Beyotime, C0071S) was used to detect the proliferation rate of GCs under BHBA stress. GCs were cultured in confocal dishes and proliferating cells were labeled with EdU at a final concentration of 10 μ*M* (1×). Then, the cells were fixed and permeabilized using 4% paraformaldehyde and PBS containing 0.3% Triton X-100, respectively, for 15 min. Click additive solution was then prepared according to the manufacturer's recommendations and added to the dishes. The GCs were then incubated at room temperature and protected from light for 30 min. After staining, the GCs were washed three times with PBS containing 3% BSA. To calculate the percentage of cell proliferation, the nuclei of GCs were stained using Hoechst 33342. Finally, fluorescence was observed using a confocal microscope. The results were analyzed by Image J software.

### Cell Cycle Detection

At the end of the BHBA treatment, GCs were collected with 0.25 % trypsin and then washed with pre-chilled PBS. Next, 1 ml of pre-chilled 70 % ethanol was added to fix the cells for 2 h at 4°C. The cells were then collected by centrifugation at 1,000 g for 5 min. A Cell Cycle and Apoptosis Assay Kit (Beyotime, C1052) was used for this experiment. Propidium iodide (PI) staining solution was prepared according to the manufacturer's instructions; then 0.5 ml of PI was added to each tube of cell samples. The GCs were then incubated for 30 mins in the dark at 37 °C. Finally, red fluorescence was detected by flow cytometry at an excitation wavelength of 488 nm. The results were analyzed by FlowJo software (version, 10.4).

### RNA Isolation and Sequencing

Three samples from each group were used for RNA extraction and library preparation. Total RNA was extracted from GCs using Trizol reagent (Invitrogen, Carlsbad, CA, USA) in accordance with the manufacturer's protocol. The quality of the RNA was assessed by an Agilent 2100 Bioanalyzer (Agilent Technology, Palo Alto, CA, USA) and detected by RNase-free agarose gel electrophoresis. After extracting total RNA from the GCs, we enriched the eukaryotic mRNA with oligo (DT) beads. Prokaryotic mRNA was enriched by removing rRNA with a Ribo-Zero™ Magnetic Kit (Epicentre, Madison, WI, USA). The enriched mRNA fragments were then cut into short fragments with fragment buffer and then reverse transcribed into cDNA with random primers. Second-strand cDNAs were synthesized by RNase H, dNTP, DNA polymerase I, and buffer. Then the cDNA fragments were purified with a QiaQuick PCR extraction kit (Qiagen, Venlo, The Netherlands), end-repaired, and a poly (A) tail was added. Finally, we added an adapter for Illumina sequencing. The ligation products were size selected by agarose gel electrophoresis, PCR amplified, and sequenced using Illumina HiSeq2500 by Gene Denovo Biotechnology Co. (Guangzhou, China). Meanwhile, the raw sequencing data were deposited in the Genome Sequence Archive in Beijing Institute of Genomics (BIG) Data Center (https://bigd.big.ac.cn/), Chinese Academy of Sciences, under the accession number: CRA006622.

### Bioinformatics and Statistical Analysis

Raw reads from adapters or low-quality bases would affect subsequent assembly and analysis. Therefore, to obtain high-quality clean reads, the reads were further filtered by FASTP (22) (version 0.18.0). The following three parameters were used to filter the raw reads: (1) removing reads containing adapters; (2) removing reads containing more than 10% of unknown nucleotides (N); and 3) removing low quality reads containing more than 50% of low quality (Q-value ≤ 20) bases. The short reads alignment tool Bowtie2 (23) (version 2.2.8) was used for mapping reads to a ribosomal RNA (rRNA) database. The mapped rRNA reads were removed and the remaining clean reads were used for assembly and gene expression calculation. The mapped reads of each sample were assembled by StringTie v1.3.1 (24, 25) using a reference-based approach. The fragment per kilobase of transcript per million mapped reads (FPKM) value of each transcription region was calculated by StringTie software to quantify the expression level.

The differential expression of RNAs between the two different groups was analyzed by DESeq2 (26) software. Genes with a *P* < 0.05 (there was no correction for multiple comparisons) and an absolute fold change (FC) > 1.5 were defined as differentially expressed genes (DEGs). To determine the patterns of gene expression in association with BHBA concentration, we performed a series of tests in clusters using Short Time-series Expression Miner (STEM) version 1.3.8.

Gene Ontology (GO) enrichment analysis was used to identify the GO terms that were significantly enriched in DEGs when comparing the genomic backgrounds. Then, we filtered the DEGs that corresponded to biological functions. All DEGs were mapped to GO terms in the Gene Ontology database (http://www.geneontology.org/) and gene numbers were calculated for every term; significantly enriched GO terms for DEGs identified between different genomic backgrounds were defined by the hypergeometric test. Meanwhile, pathway-based analysis was used to further elucidate the biological function of specific genes; for this part of our analysis, we used a public pathway-related database (27) based on the Kyoto Encyclopedia of Genes and Genomes (KEGG) (28). GO terms and pathways for which *P* < 0.05 were considered to represent significantly enriched pathways.

### Validation of RNA-Seq Data

Next, we used quantitative real-time polymerase chain reaction (qRT-PCR) to verify the RNA-seq results. Total RNA was extracted from the GCs as described in the previous steps. Then, the RNA (1 μg) was reverse transcribed into cDNA by using PrimeScript™ RT reagent Kit with gDNA Eraser (RR047A, Takara) according to the manufacturer's instructions. Gene expression levels in GCs were then detected using PowerUp™ SYBR™ Green Master Mix (A25742, ABI) and a QuantStudio™ 7 Flex System (ABI); each sample was analyzed in triplicate. We used the house-keeping gene *GAPDH* as a reference and the expression levels of each gene were calculated by the 2^−Δ*ΔCT*^ method. Reactions for qRT-PCR were set up as follows: in a total volume of 15 μL: SYBR Green master mix, 7.5 μL; forwarding primer, 0.5 μL; reverse primer, 0.5 μL; cDNA, 2 μL; and ddH_2_O, 4.5 μL) with specific cycle parameters (50°C, 2 min; 95°C, 2 min; followed by 40 cycles at 95°C for 1 s and 60°C for 30 s) as stated in the manufacturer's instructions. All primers used for qRT-PCR were designed by the National Center for Biotechnology Information (NCBI); sequences are shown in Table 1 and synthesized by The Beijing Genomics Institute (BGI).

**Table 1:** Primers used for the qRT-PCR validation of RNA-seq results.

**Gene target**	**NCBI sequence**	**Primer Sequence (5^**′**^ → 3^**′**^)**	**Product size (bp)**
*EGR1*	NM_001045875.1	F: ACCTGACCGCAGAGTCCTTT	150
		R: AAGTGTTGCCACTGTTGGGT	
*OAS1X*	NM_178108.2	F: TGAAGCTCATCCAAGAGTGCG	161
		R: CTTATGCTTCATCTTACACAGTTGG	
*OAS1Y*	NM_001040606.1	F: GACGCAAATAGCTGGGAGCG	118
		R: TGTGTTCTTGGGGCGACACAT	
*ANKRD1*	NM_001034378.2	F: CTCAATGCCAAAGACCGGGA	131
		R: GGAGTCTTCCCAGCACAGTTC	
*VCAN*	NM_181035.2	F: TGATGCCTACTGCTTTAAACCTAA	129
		R: GGTGTCATTCTGTCCAGTCCC	
*FST*	NM_175801.3	F: TGAGCTGTGCCCTGAGAGTA	158
		R: CGGTGTCTTCCGAAATGGAGTT	
*NREP*	NM_001105045.1	F: GGGGCTTTTGTCTGTTGGTT	191
		R: GGTTCACTTCCTTGGGGACA	
*STAR*	NM_174189.3	F: ACGTCAAGGAGATCAAGGTCC	113
		R: TACGCTCACAAAGTCTCGGG	
*CYP26B1*	NM_001192793.2	F: GGAACAAGCGCAAGGTCTTC	136
		R: TCCTGGTACACGTTGATGGC	
*FOS*	NM_182786.2	F: AAAAGGCGAATCCGAAGGGA	102
		R: TAGTTGGTCTGTCTCCGCTTG	
*FOSB*	NM_001102248.1	F: GAGAAGAGAAGGGTTCGCCG	106
		R: CTAGCTGATCTGTCTCCGCC	
*LSS*	NM_001046564.1	F: ATCCTGTCCGAGAGCAGTCT	204
		R: TGATCAGGAGGCCTGGCA	
*GAPDH*	NM_001034034	F: GGGTCATCATCTCTGCACCT	177
		R: GGTCATAAGTCCCTCCACGA	

### Statistical Analysis

We used SAS software version 9.2 for statistical analysis. Groups were compared by one-way analysis of variance (ANOVA) followed by the Duncan *post-hoc* test for multiple comparisons. *P* < 0.05 was considered statistically significant. Results are shown as means ± standard deviation (SD). All experiments were repeated at least in three independent experiments.

## Results

### The Effects of BHBA Exposure on the Viability of GCs

**Figure 1:**
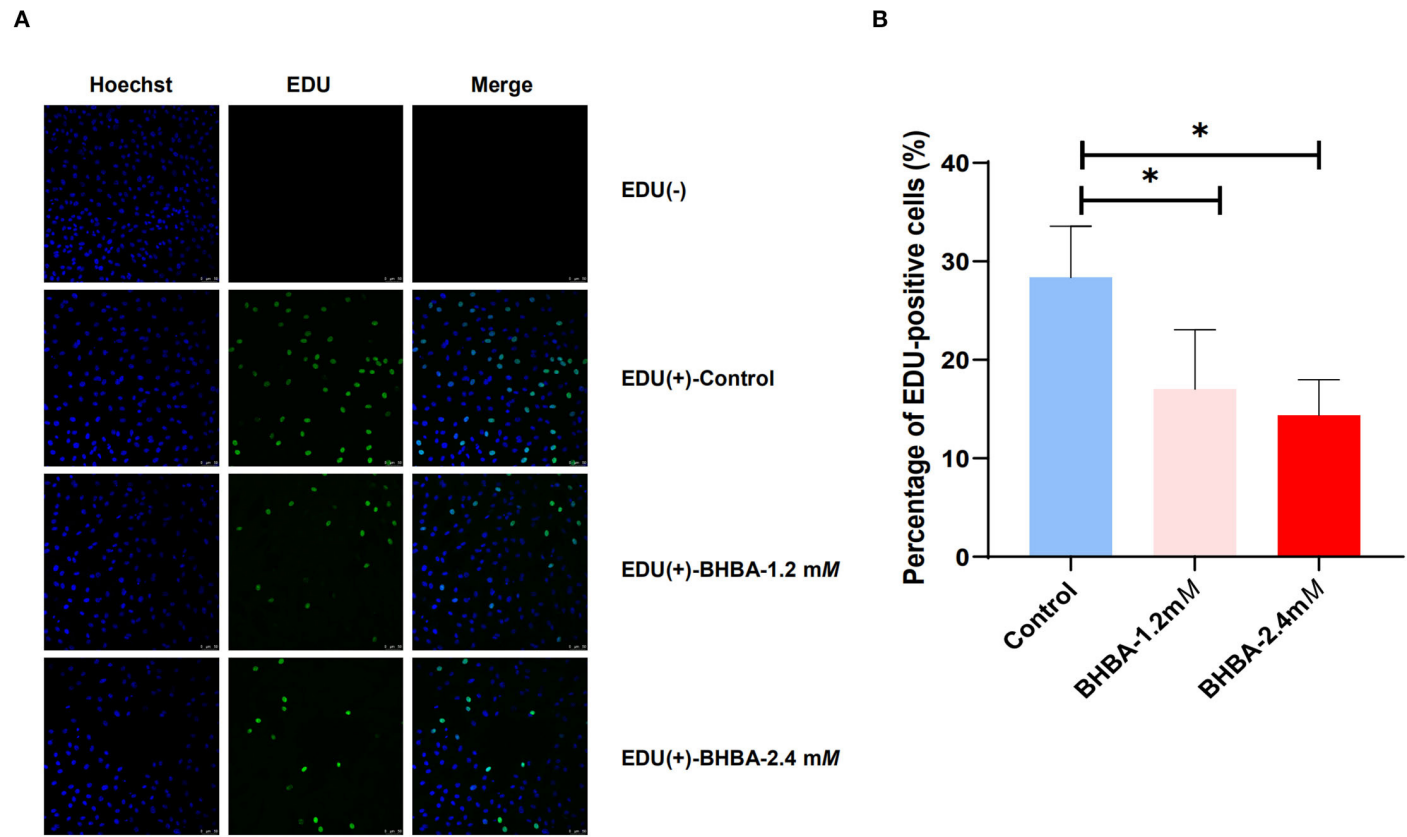
The effect of different concentrations of BHBA stimulation on the proliferation rate of GCs. **(A)** The proliferation of GCs was detected by EdU. Cell nuclei were stained blue by Hoechst 33342. **(B)** EdU-positive cells (shown in green) were counted and the percentage was calculated. The bar graph shows the mean ± SD of each group, *P < 0.05.

We used the EdU assay to investigate the effect of increasing concentrations of BHBA on the proliferation of GCs. As shown in Figure 1, we calculated the percentage of EdU-positive cells in each of the three groups by cell counting; the EdU (-) group was the negative control. Compared to the control group, the proliferation rate of GCs in the BHBA-1.2 m*M* and BHBA-2.4 m*M* groups were significantly lower (*P* < 0.05). However, the proliferation rate was lower in the BHBA-2.4 m*M* group than in the BHBA-1.2 m*M* group, although this was not statistically significant (*P* > 0.05). These results indicate that the concentration of BHBA was negatively correlated with the proliferation rate of GCs.

### The Effect of BHBA on the Cell Cycle in GCs

**Figure 2:**
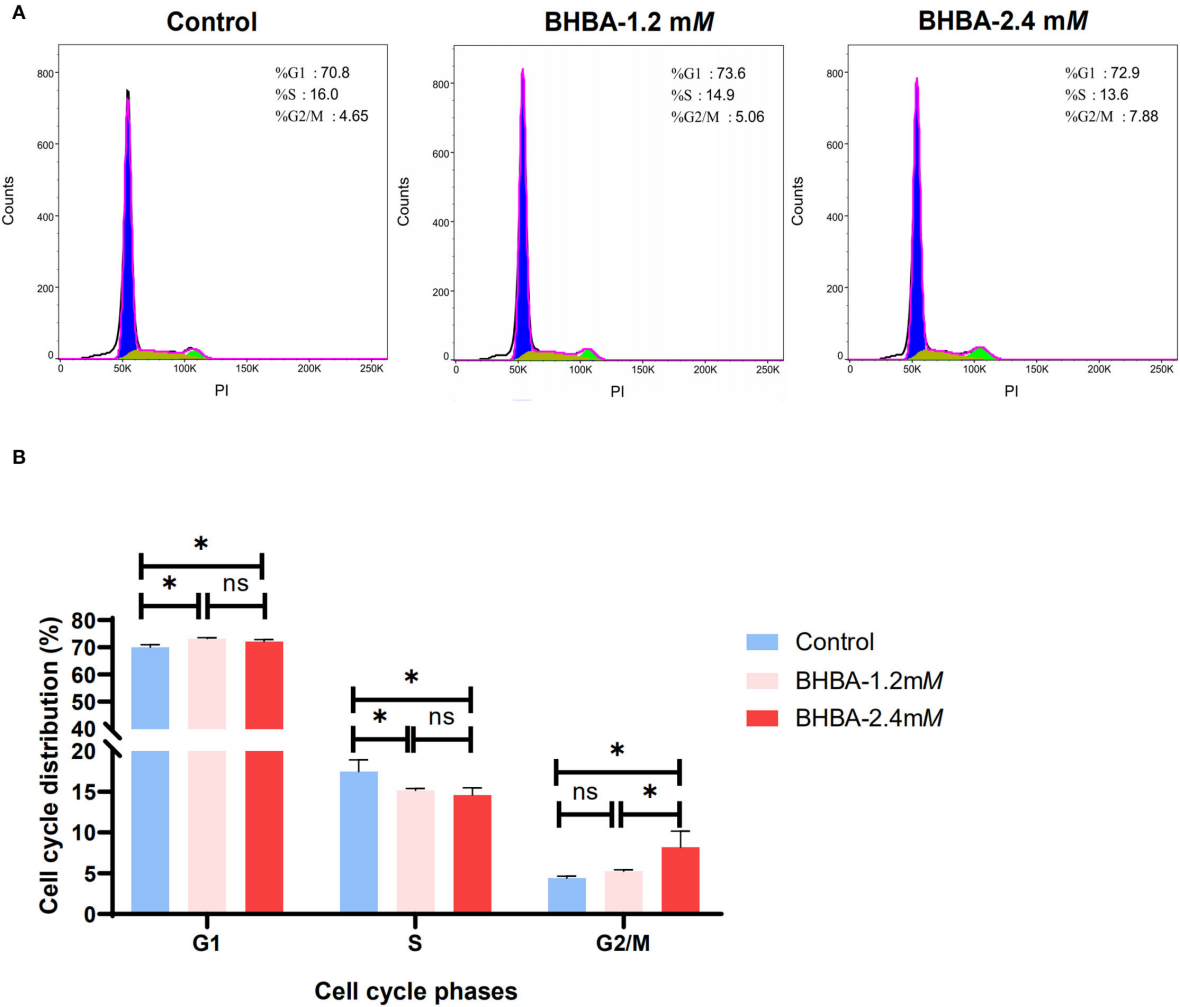
Effect of different concentrations of BHBA stimulation on the cell cycle of GCs. **(A)** Flow cytometry analysis of the cell cycle phase distribution of GCs under different concentrations of BHBA stimulation. **(B)** Bar graph showing the mean ± SD of each group, compared to the control group, *P < 0.05, ns represents *P* > 0.05.

Next, we tested whether the BHBA-induced inhibition of proliferation in GCs was caused by a disruption in the cell cycle; for this, we used flow cytometry. Compared to the control group, the percentage of GCs in G1 phase was significantly higher in the BHBA-1.2 m*M* and BHBA-2.4 m*M* groups (*P* < 0.05). In contrast, the percentage of GCs in S phase was significantly lower (*P* > 0.05) (Figure 2). These results indicated that both 1.2 m*M* and 2.4 m*M* BHBA induced cell cycle arrest in the G1 phase. In addition, we observed that the number of GCs in the G2/M phase was significantly higher in the BHBA-2.4 m*M* group than in the control and BHBA-1.2 m*M* groups (Figure 2). Consequently, the clear disruption in the cell cycle may be related to a concomitant reduction in the proliferation of GCs.

### Overview of RNA-Seq Data

To demonstrate the changes in gene expression patterns of GCs treated with different concentrations of BHBA, we used RNA-seq to determine the transcriptome profiles of the three groups (control, BHBA-1.2 m*M*, and BHBA-2.4 m*M*) in GCs. Clean reads were aligned to the *Bos taurus genome* after discarding raw data. The percentage of clean reads with Q30 in all samples ranged from 91 to 92%, and the percentage of total mapped reads was approximately 97%; the unique mapped rate was also approximately 95% (Table 2). Taken together, these results indicated that the RNA-seq data were of high quality and could support subsequent analysis.

**Table 2:** RNA-seq data quality analysis.

**Sample**	**Raw data-reads**	**Clean data-reads (%)**	**Raw data-bases (bp)**	**Clean data- bases (bp)**	**Q30(%)**	**Unique mapped (%)**	**Total mapped (%)**
Control-1	53163632	52954650 (99.61%)	7974544800	7906362318	91.74%	95.02%	97.05%
Control-2	58226504	57961884 (99.55%)	8733975600	8649545545	91.73%	94.88%	96.90%
Control-3	55371514	55113446 (99.53%)	8305727100	8213218256	91.66%	94.82%	96.88%
BHBA-1.2 m*M*-1	55728944	55534290 (99.65%)	8359341600	8286852601	91.87%	94.99%	96.97%
BHBA-1.2 m*M*-2	57007148	56790872 (99.62%)	8551072200	8479498744	91.53%	94.81%	96.83%
BHBA-1.2 m*M*-3	57612176	57386606 (99.61%)	8641826400	8565037565	91.65%	94.88%	96.87%
BHBA-2.4 m*M*-1	50080934	49913860 (99.67%)	7512140100	7452526902	91.78%	95.13%	97.17%
BHBA-2.4 m*M*-2	51265612	51072296 (99.62%)	7689841800	7616129077	91.69%	94.87%	96.92%
BHBA-2.4 m*M*-3	60008924	59767000 (99.60%)	9001338600	8913626489	91.62%	94.82%	96.87%

### Analysis of Differentially Expressed Genes

**Figure 3:**
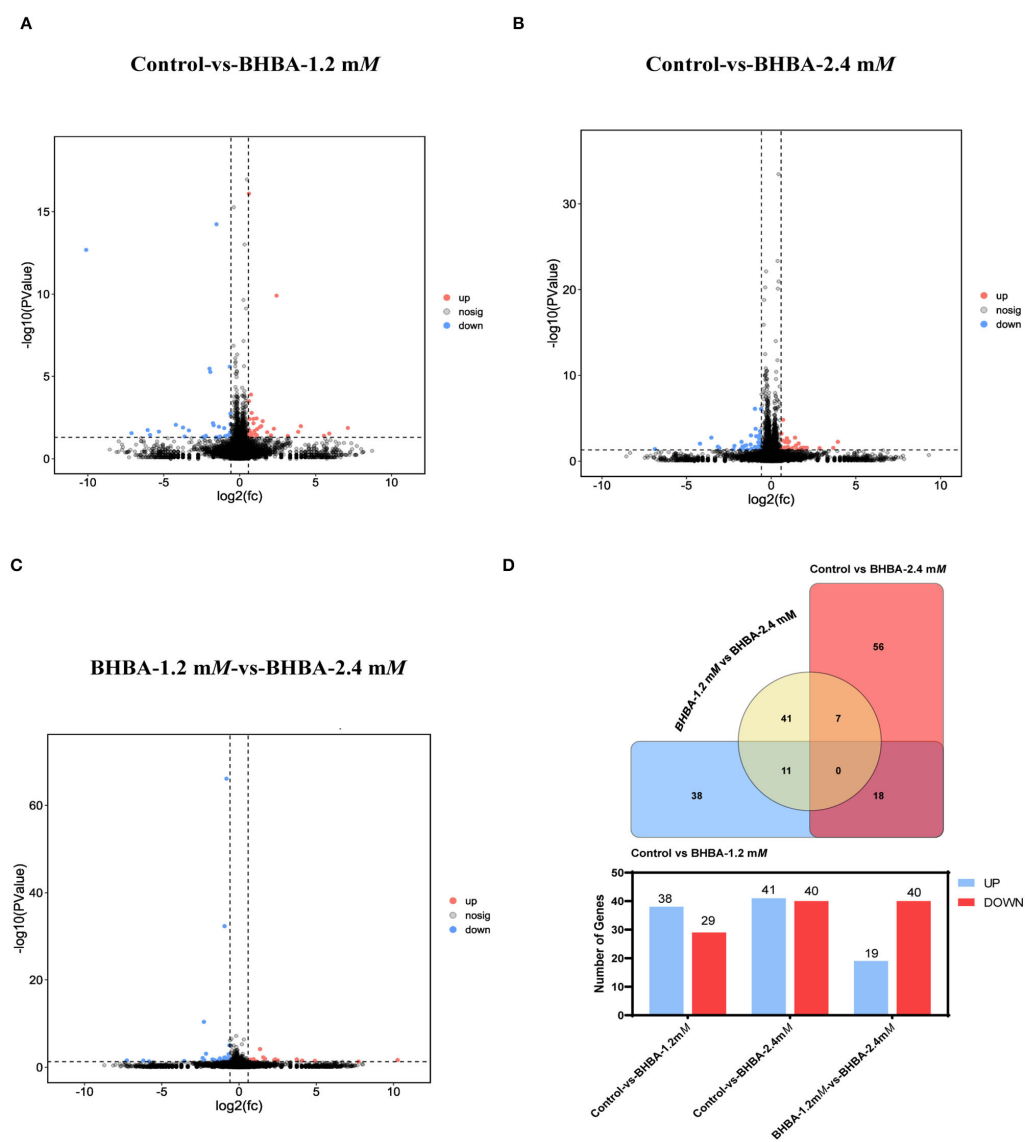
Significant DEGs were identified between the three groups. The screening criteria for significant DEGs was *P* < 0.05 and |FC| > 1.5. **(A–C)** show volcanic plots of the significant DEGs across the three groups. **(D)** The Venn diagram shows the common and specific DEGs in the three comparison groups. And the bar chart shows the number of significant DEGs in the three groups.

To investigate the effects of different concentrations of BHBA on the patterns of gene expression in GCs, we identified DEGs between the different BHBA treatment groups based on *P* < 0.05 and |FC| > 1.5. As shown in Figure 3, gene expression differed significantly across the three groups. A total of 67 significant DEGs were identified when comparing the Control group with the BHBA-1.2 m*M* group, including 38 up-regulated genes and 29 down-regulated genes (Figures 3A,D, and Supplementary Table 1). The numbers of up-regulated and down-regulated genes of the 81 significant DEGs identified when comparing the Control group with the BHBA-2.4 m*M* group were similar (41 and 40, respectively) (Figures 3B,D, and Supplementary Table 1). In addition, we identified 59 significant DEGs when comparing the BHBA-1.2 m*M* group and the BHBA-2.4 m*M* group; the number of up-regulated genes was almost half the number of down-regulated genes (19 and 40, respectively) (Figures 3C,D, and Supplementary Table 1).

### Gene Expression Pattern Analysis and Clustering

**Figure 4:**
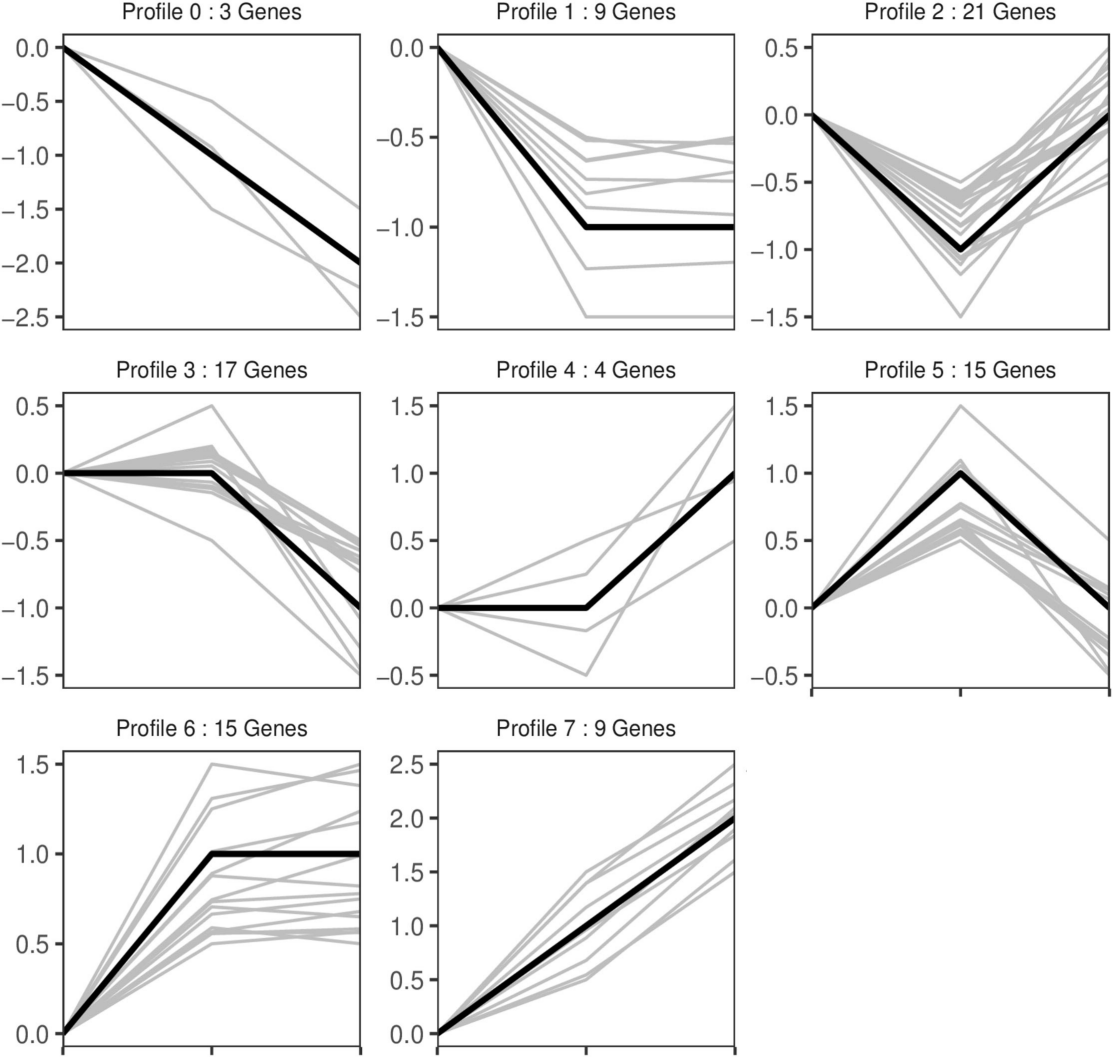
The expression patterns of DEGs were analyzed. Each node in the profile is Control, BHBA-1.2 m*M*, and 2.4 m*M* from left to right respectively.

Trend analysis is used to classify genes with similar expression patterns. We used trend analysis to generate representative clusters for different concentrations of BHBA, thus revealing patterns that were specific to GCs during BHBA stimulation (29). We performed a series of clustering experiments on DEGs. Short Time-series Expression Miner (STEM) and log2 normalized preprocessed data were used to analyze the expression patterns of DEGs in the BHBA treatment groups at 0 m*M*, 1.2 m*M*, and 2.4 m*M*. The DEGs from the three comparison groups were clustered into eight profiles (Figure 4). We classified the profiles into three types of clusters, including low-concentration (LC) responders (profile 1 and profile 6), high-concentration (HC) responders (profile 3 and profile 4), and the similar trend (ST) responders (up or down) following BHBA concentrations (profile 0 and profile 7), respectively. We found that DEGs in profiles 1 and 6 (including 15 up-regulated DEGs and 9 down-regulated DEGs) responded to 1.2 m*M* BHBA stimulation but remained constant as the 2.4m*M* BHBA stimulation (Figure 4, Supplementary Table 2). In addition, 17 down-regulated genes and 4 up-regulated genes were clustered in profiles 3 and 4, thus demonstrating a response to high concentrations (2.4 m*M*) of BHBA (Figure 4, Supplementary Table 2). Finally, DEGs in profile 0 and profile 7 showed an ST effect under increasing concentrations of BHBA, including 3 down-regulated DEGs and 9 up-regulated DEGs (Figure 4, Supplementary Table 2).

### Functional Enrichment of Differentially Expressed Genes

**Figure 5:**
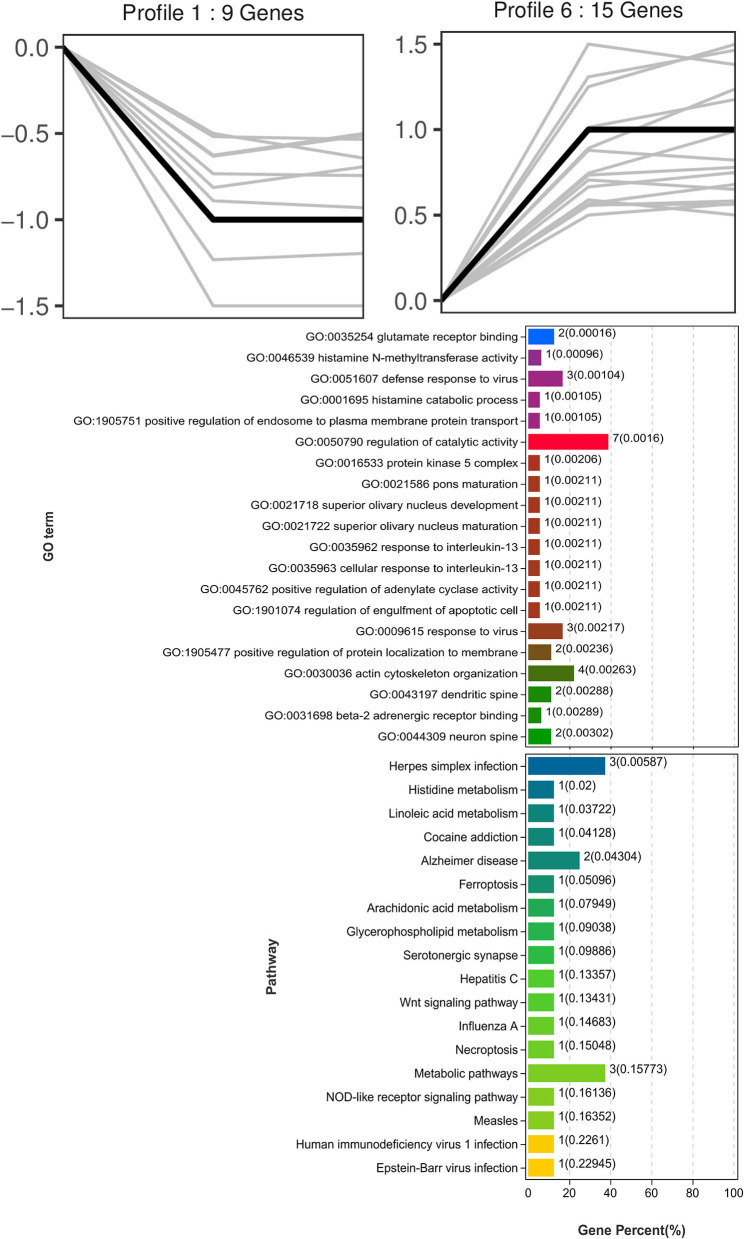
GO and KEGG enrichment analysis of DEGs (in profile 1 and profile 6) with the LC response at BHBA-1.2 m*M* concentration.

**Figure 6:**
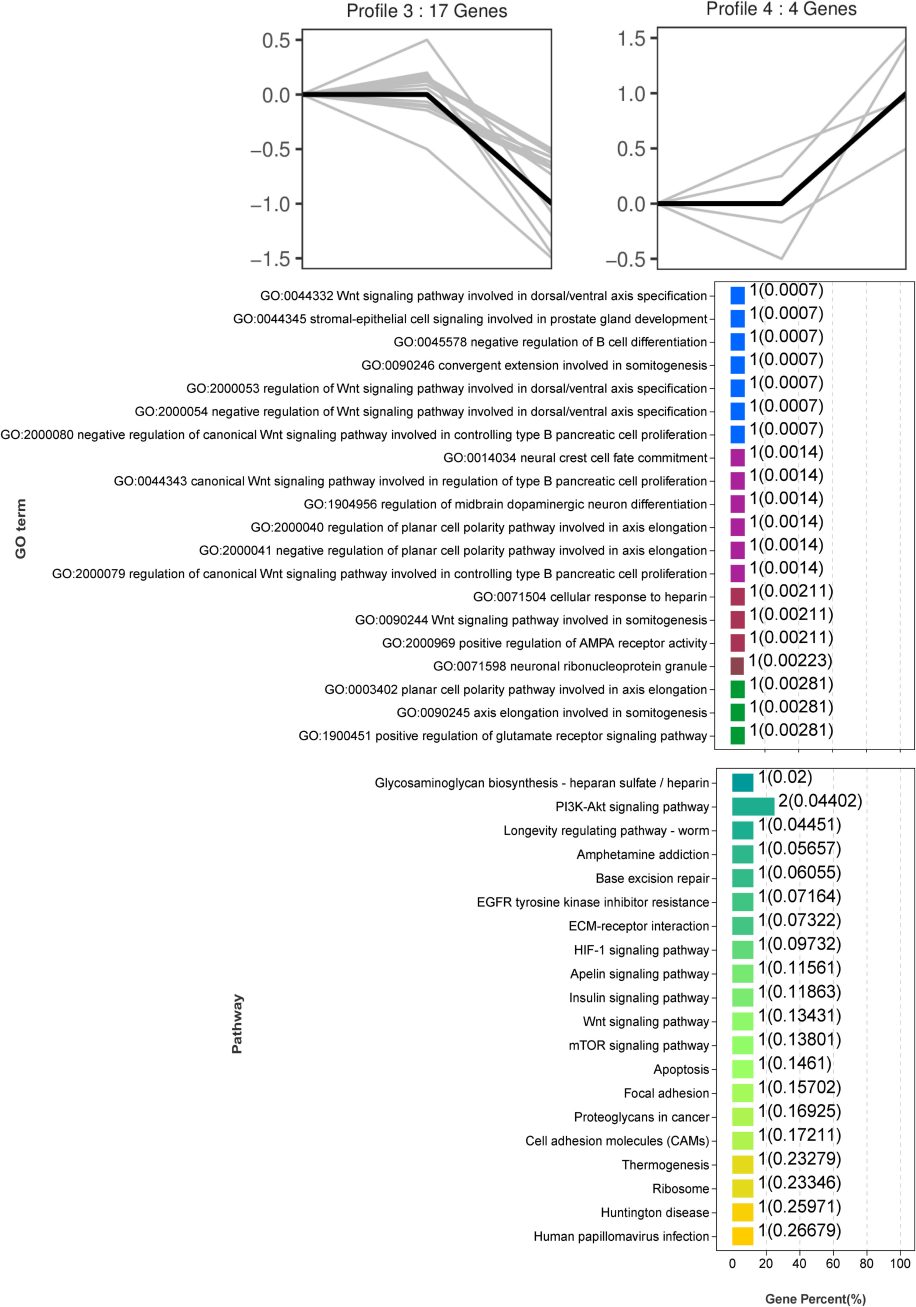
GO and KEGG enrichment analysis of DEGs (in profile 3 and profile 4) with the HC response at BHBA-2.4 m*M* concentration.

**Figure 7:**
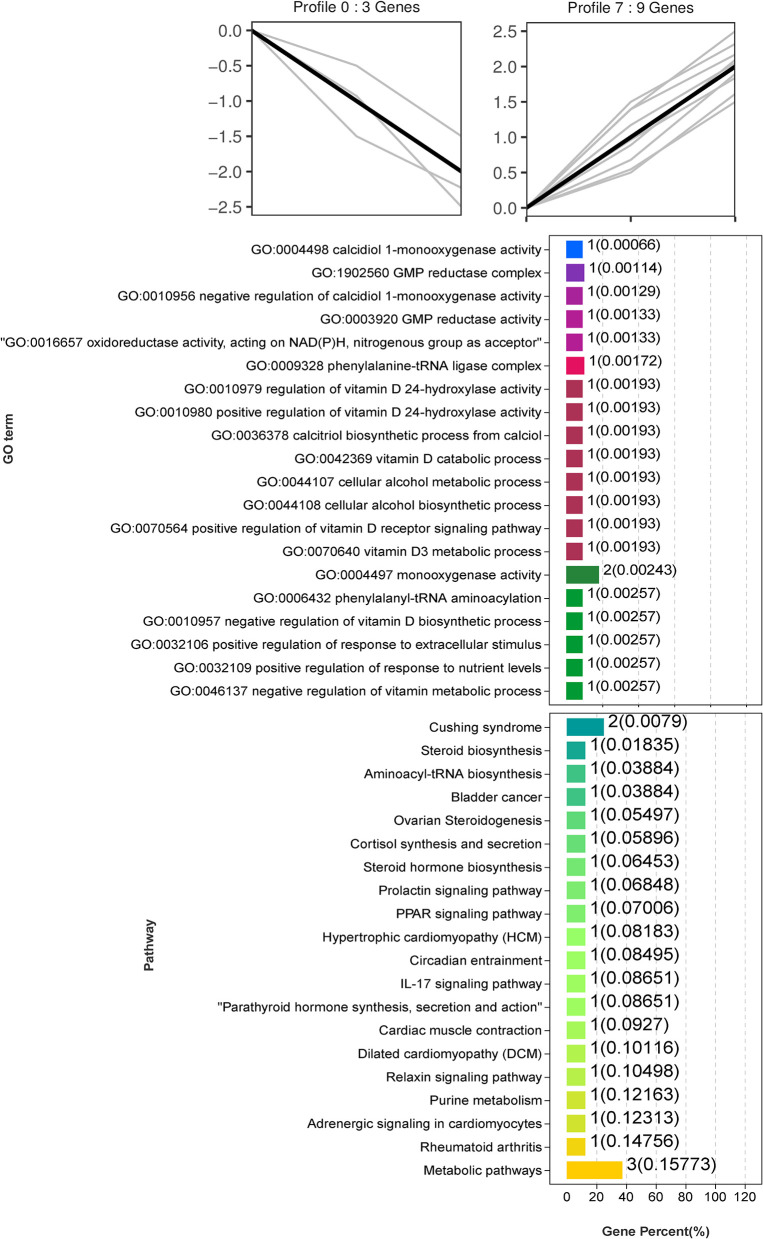
GO and KEGG enrichment analysis of the ST responding DEGs (in profile 0 and profile 7) with increasing BHBA concentrations.

To further investigate the function of DEGs in different response modes, we performed GO and KEGG enrichment analysis of the DEGs in three modes (LC response, HC response, and ST response) under different concentrations of BHBA. We found that the most significantly enriched pathway (ranked by *p*-value) for the LC response cluster was Herpes simplex infection; other significant GO terms were involved in the immune response, such as defense response to virus and response to interleukin-13 (Figure 5). These DEGs responded when exposed to 1.2 m*M* concentrations of BHBA, thus demonstrating that these genes play a key role in the LC of BHBA stress. We also found that DEGs involving signal transduction and the apoptosis pathway were enriched in profile 3 and profile 4 (Figure 6). These genes were insensitive at HC of BHBA stress; up- or down-regulation occurred only when 2.4 m*M* BHBA was stimulated, thus demonstrating that these genes drove an HC response to BHBA stimulation. DEGs involved in the endocrine system and metabolism were enriched in profile 0 and profile 7 (Figure 7). These DEGs showed an ST response to increased BHBA stress.

### Validation of RNA Sequencing by qRT-PCR

**Figure 8:**
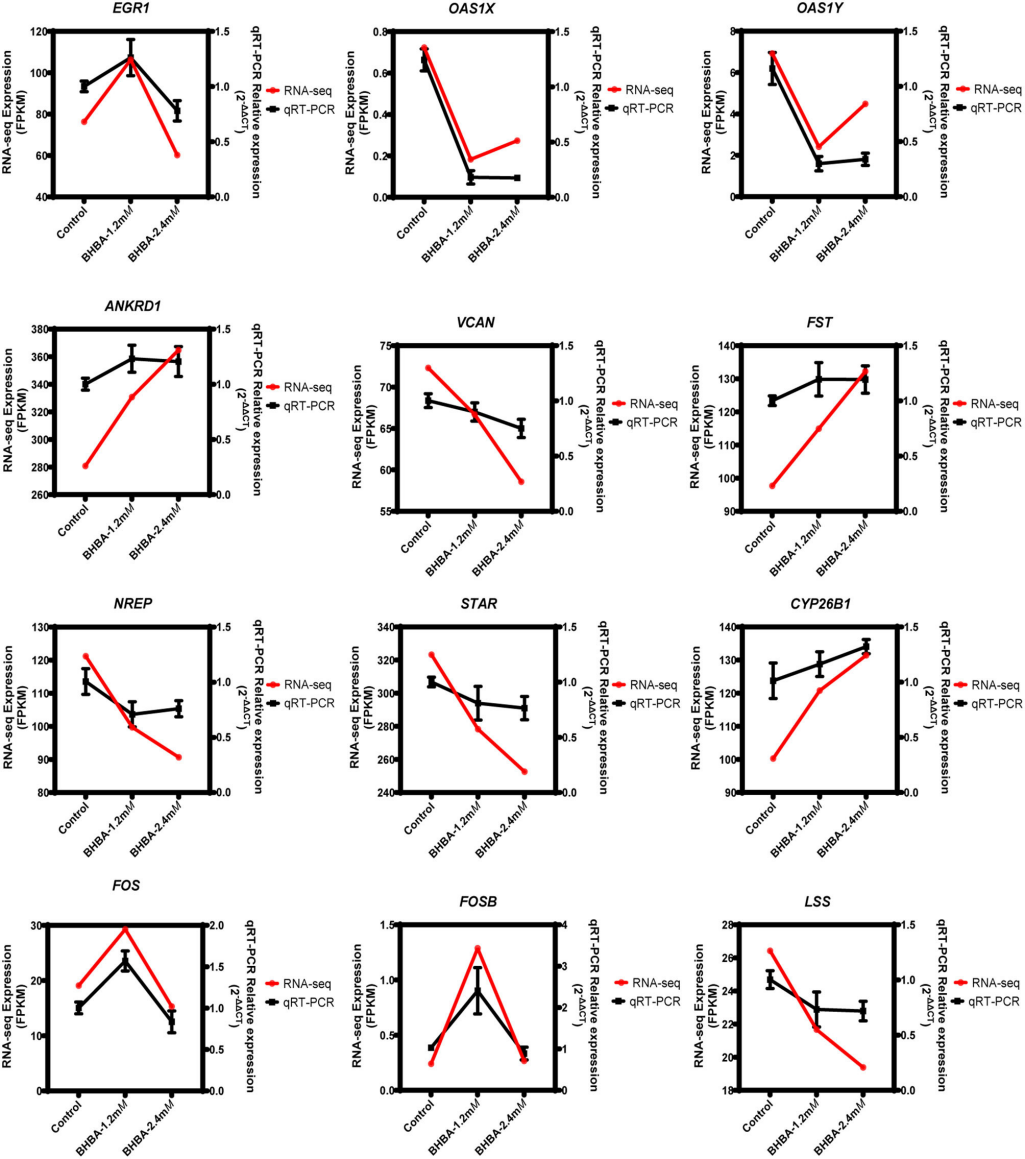
Validation of the RNA-seq data by qRT-PCR.

To demonstrate the accuracy of the RNA-seq data, we randomly detected the expression levels of 12 genes (including *EGR1, OAS1X, OAS1Y, ANKRD1, VCAN, FST, NREP, STAR, CYP26B1, FOS, FOSB*, and *LSS*) by qRT-PCR. As shown in Figure 8, the RNA-seq results were consistent with the expression levels generated by qRT-PCR. These results clearly demonstrated the reliability of the RNA-seq data generated in this study.

## Discussion

During early lactation, the DMI of dairy cows is not sufficient to meet their total energy requirements. Therefore, fat is mobilized to keep up with the demands of lactose synthesis, thus resulting in a significant increase in KBs (indicated by high levels of BHBA). Cows in this condition, especially those with a high yield, are predisposed to NEB and ketosis (30–32). Originally, some increase in the amount of KBs was considered to be a natural metabolic response and these KBs could even act as an energy carrier to meet energy demand under certain physiological conditions, including starvation (5). Hence, low doses of BHBA have been shown to exert positive effects in the treatment of certain diseases (33, 34). However, if the concentration of BHBA increases excessively, this could directly or indirectly affect normal liver metabolism (35, 36) and reproductive performance (e.g., granulosa cell development and oocyte maturation) (20, 37, 38) in ruminants such as cows.

The molecular regulatory mechanisms of ketosis-induced reproductive disturbances in cows remain unclear. The normal development of GCs is necessary to ensure a normal follicular cycle and maintaining the balance between the proliferation and apoptosis of GCs is vitally important for oocyte maturation and embryonic development (39). When this balance is disturbed, follicular development will be either retarded or even terminated (40). It is generally considered that *in vitro* cell models are particularly valuable for studying steroidogenesis and folliculogenesis, especially for humans and large ruminants (41, 42). Although *in vitro* cell models cannot completely simulate the complex regulatory environment *in vivo*, their contribution to the exploration of some complex disease mechanisms should not be ignored because of their convenience, low cost, easy availability, and focus (43–46). In this study, we established an *in vitro* model of GCs and found that as the concentration of BHBA increased, the rate of GC proliferation decreased significantly (Figure 1). In addition, cell cycle assays showed that BHBA-induced GCs arrested in the G1 phase (Figure 2), thus suggesting that BHBA may inhibit cell proliferation by affecting the cell cycle. This is consistent with previous findings in SCK cows, in that blocking the proliferation of GCs may be a key factor underlying abnormalities in the follicular cycle and estrus cycle, thus reducing reproductive performance (10, 12, 47).

Therefore, in this study, we aimed to explore changes in gene expressions under different concentrations of BHBA by using *in vitro* cellular models and RNA-seq technology. First, we identified 67, 81, and 59 DEGs when comparing three treatment groups (including Control *vs* BHBA-1.2 m*M*, Control *vs* BHBA-2.4 m*M*, and BHBA-1.2 m*M vs* BHBA-2.4 m*M*) and used these DEGs for trend analysis (Figure 3). These DEGs were clustered into eight gene profiles and three expression clusters, including the LC responding genes (the expression of DEGs peaked in the BHBA-1.2 m*M* group, profiles 1 and 6), the HC responding genes (the expression of DEGs peaked in the BHBA-2.4 m*M* group, profiles 3 and 4) and the ST responding genes (the continuous expression of DEGs along with increasing concentrations BHBA, profiles 0 and 7) (Figure 4). Finally, we performed GO and KEGG analyses for DEGs in each different cluster and then described the function of the DEGs in response to different concentrations of BHBA. Our analysis yielded a dynamic regulatory network transcriptional profile that showed constant changes as a result of increasing concentrations of BHBA.

**Figure 9:**
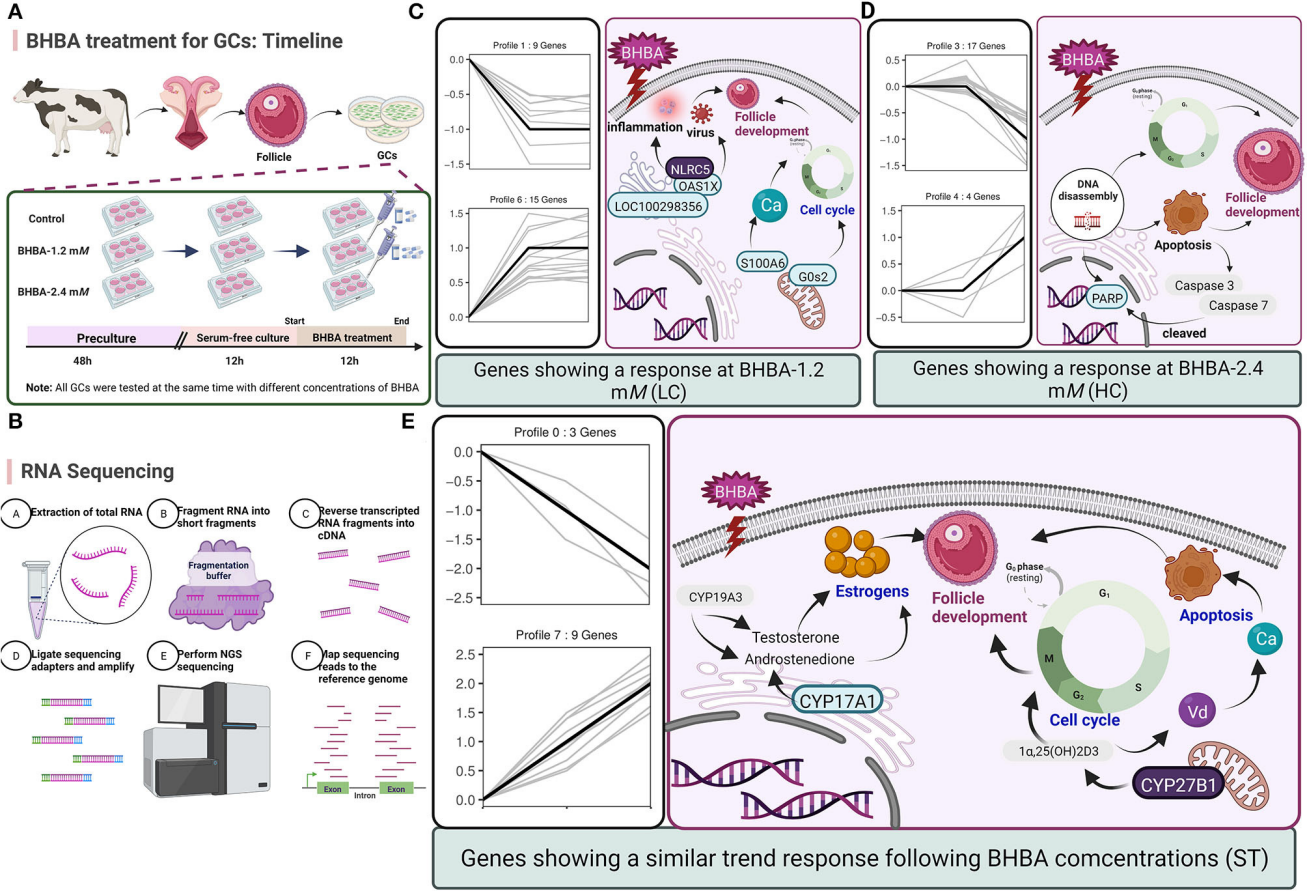
**(A)** Timeline of BHBA treatment for GCs. **(B)** The process used for RNA-seq. **(C–E)** Predicting the regulatory effects on GCs and follicle development exerted by the major DEGs in three expression patterns (including LC response, HC response, and ST response). Up-regulated DEGs are shown in blue boxes while down-regulated DEGs are shown in gray boxes.

Next, we investigated the functions of the LC responding genes; the most significantly enriched pathway was herpes simplex infection (including *OAS1X, LOC100299025*, and *LOC100298356*). Several other immune response-related GO terms, such as defense response to virus and response to interleukin-13, were also significantly enriched. Similarly, SCK cows have been reported to show a raised immune and inflammatory response around the time of delivery (48–50). However, under an excessive inflammatory environment, the development of GCs and follicles may be restricted, thus leading to ovulatory dysfunction (51). Notably, in the LC responding cluster, we identified two novel down-regulated genes (*G0s2* and *S100A6*) that play a role in the regulation of cell proliferation and cycle progression. Of these, *G0S2* is defined as G0/G1 switch gene 2 and is known to be essential for cell cycle regulation (52, 53). *S100A6* was also down-regulated and belongs to the family of calcium-binding proteins; this gene is also known as calcyclin and can translate Ca^2+^ signals into specific actions such as cell cycle activity (54, 55). Therefore, we hypothesized that BHBA-induced G1 phase arrest and the reduced proliferation rate of GCs correlated with the down-regulation of *G0S2* and *S100A6* (Figure 9). A similar study previously showed that BHBA could block the cycle of bovine abomasum smooth muscle cells (BSMCs) in the G1 phase by inactivating cyclins/CDKs; these authors also reported that BHBA may induce apoptosis by increasing intracellular levels of Ca^2+^ (17).

With respect to the HC responding genes, we were surprised to identify a critical down-regulated DEG (*PARP*) that was enriched in the apoptosis pathway and may be sensitive to higher concentrations of BHBA (2.4 m*M*). PARP is an enzyme that plays a role in genome monitoring and DNA repair. This enzyme is recognized by activated caspase −3 and caspase −7 when DNA damage is severe enough to cause apoptosis. The DEVD motif of PARP is recognized by caspase proteases and cleaved into two fragments (P89 and P24), thus resulting in its inactivation (56). When cellular DNA is damaged, the cell cycle normally arrests in the G2/M phase to allow repair; if the damage is irreparable, the apoptotic pathway can be activated (57). In the present study, the expression of *PARP* in GCs was significantly reduced in the BHBA-2.4 m*M* group; in addition, GCs were blocked in the G2/M phase. These results indicated that high levels of BHBA may lead to DNA damage and the activation of apoptosis in GCs. Collectively, this evidence suggested that stimulation with increasing concentrations of BHBA (1.2 m*M*) in SCK cows initiated an adaptive response in GCs undergoing cell cycle disruption. Furthermore, when higher concentrations of BHBA trigger CK in cows, it may cause irreparable DNA damage and induce apoptosis in GCs (Figure 9). This might explain the impaired follicular development and reduced reproductive performance of cows with SCK and CK.

We also identified several DEGs that showed an ST response to increased levels of BHBA, thus helping us to better characterize the reaction of the GCs. We identified an important up-regulated gene *CYP27B1* that was enriched in the most significant GO terms (calcidiol 1-monooxygenase activity) and other multiple vitamin D-regulated GO terms. CYP27B1 is a mitochondrial cytochrome P450 that catalyzes the hydroxylation of 25OHD3 (25-hydroxyvitamin D3, calcifediol) at the C1α position to produce the biologically active 1α,25(OH)2D3 (1α,25-dihydroxycholecalciferol, calcitriol) (58, 59). It is well known that vitamin D plays a key role in regulating calcium and phosphorus homeostasis (60–62). Another study reported that when ketosis occurs in cows, many pathways associated with ion homeostasis are dysregulated, including the calcium pathway (63). Indeed, higher intracellular Ca^2+^ levels can promote the accumulation of ROS through the mitochondrial respiratory chain, thus inducing mitochondrial and endoplasmic reticulum stress and increasing the risk of apoptosis (17). In addition, when activated by CYP27B1, calcitriol can mediate the arrest of multiple cancer cell cycles (64–66). Therefore, we hypothesized that disruption of the cycle of GCs in response to BHBA may be related to a rise in *CYP27B1*. Interestingly, we observed a low expression level of *CYP17A1* that was enriched in the ovarian steroidogenesis pathway. *CYP17A1* is normally expressed in theca cells but may be regulated by GCs (67). Similarly, our present study identified low expression levels of *CYP17A1* in GCs; however, levels decreased progressively with increasing concentrations of BHBA. This is probably due to CYP17A1 catalyzing the production of androstenedione from progesterone, an intermediate product of estrogen synthesis regulated by *CYP19A3* (68). The level of estrogen in the follicular fluid can exert a significant effect on follicle development and is significantly associated with the DNA content of granulosa cells (69). The results of our present may imply that reduced levels of *CYP17A1* may be a factor that affects estrogen synthesis. In summary, vitamin D synthesis and DEGs that are related to estrogen synthesis give an ST response to BHBA increases in GCs, and these genes may play an essential role in the progression of ketosis in cows (Figure 9).

## Conclusion

**Figure 10:**
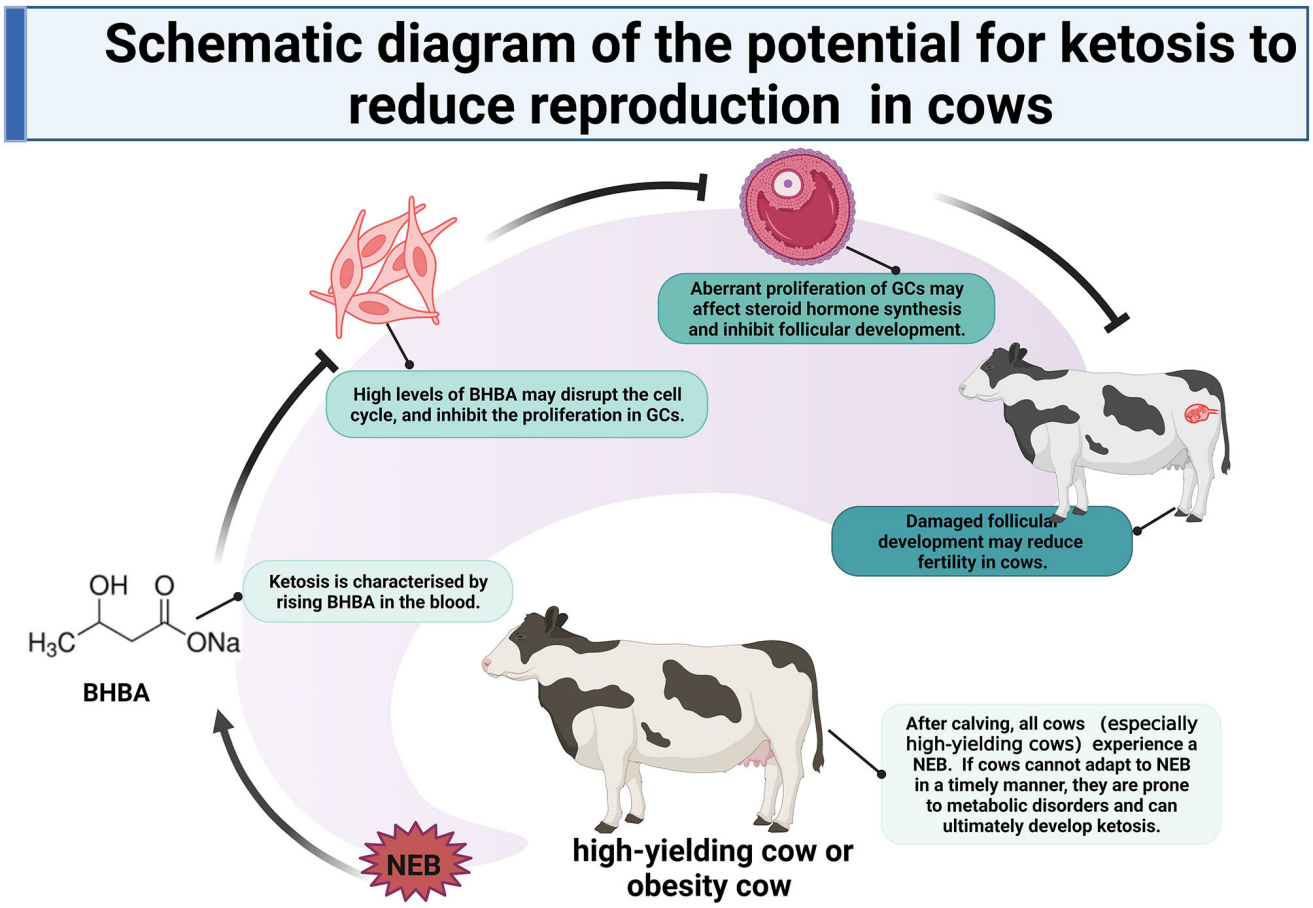
Schematic diagram of the potential for ketosis to reduce reproduction in cows.

In summary, we compared the transcriptomic dynamics of LC responders, HC responders, and ST responders of bovine GCs to BHBA stress. Our results showed that (1) increasing concentrations of BHBA in SCK and CK cows may inhibit the proliferation of GCs and disrupt their cell cycle; (2) the reduced reproductive performance of SCK and CK cows may be associated with disruption of the cell cycle in GCs and apoptosis induced by BHBA increases, and (3) increased BHBA during the development of ketosis may be associated with Ca^2+^ disruption and impaired estrogen synthesis. High levels of BHBA in ketosis cows may lead to damaged follicular development and ultimately reduced fertility by inhibiting the proliferation of follicular GCs. These findings provide a key reference to uncovering the molecular mechanisms by which SCK and CK can exert negative effects on follicular development and reproductive performance in cows (Figure 10).

## Data Availability Statement

The data presented in the study are deposited in the Genome Sequence Archive in Beijing Institute of Genomics (BIG) Data Center (https://bigd.big.ac.cn/), Chinese Academy of Sciences, accession number CRA006622.

## Ethics Statement

The animal study was reviewed and approved by Institute of Animal Science Chinese Academy of Agricultural Sciences.

## Author Contributions

JG determined the phenotypic experiments, analyzed the RNA-seq data, and participated in the writing of the original draft. SZ conceptualized the study, wrote parts of the article, and improved the initial draft. NH provided personal insights into some sections and produced some figures. YW prepared the references and checked part of the article. ZH and HW provided personal insights into some sections of the article. HZ helped with the article conception and outline, helped with revisions, and checked the article before submission. All authors contributed to the article and approved the final version.

## Funding

This work was supported by the National Natural Science Foundation of China (32102549), the Fundamental Research Funds for Central Non-profit Scientific Institution (2019-YWF-YB-03), the National Key R&D Program of Ningxia (2021BEF02023), the Modern Agro-Industry Technology Research System of the PR China (CARS-36), and the Agricultural Science and Technology Innovation Program (ASTIP-IAS06).

## Conflict of Interest

The authors declare that the research was conducted in the absence of any commercial or financial relationships that could be construed as a potential conflict of interest.

## Publisher's Note

All claims expressed in this article are solely those of the authors and do not necessarily represent those of their affiliated organizations, or those of the publisher, the editors and the reviewers. Any product that may be evaluated in this article, or claim that may be made by its manufacturer, is not guaranteed or endorsed by the publisher.
