# Supplementary material for: The Dynamic Transcription Profiles of Proliferating Bovine Ovarian Granulosa When Exposed to Increased Levels of β-Hydroxybutyric Acid
Source: Front Vet Sci. 2022 Aug 5;9:915956. doi: 10.3389/fvets.2022.915956 (PMC9389329; doi:10.3389/fvets.2022.915956)
Supplement: Supplementary file 1 [file Data_Sheet_1.docx]

Supplementary Material

# Supplementary Figure

| 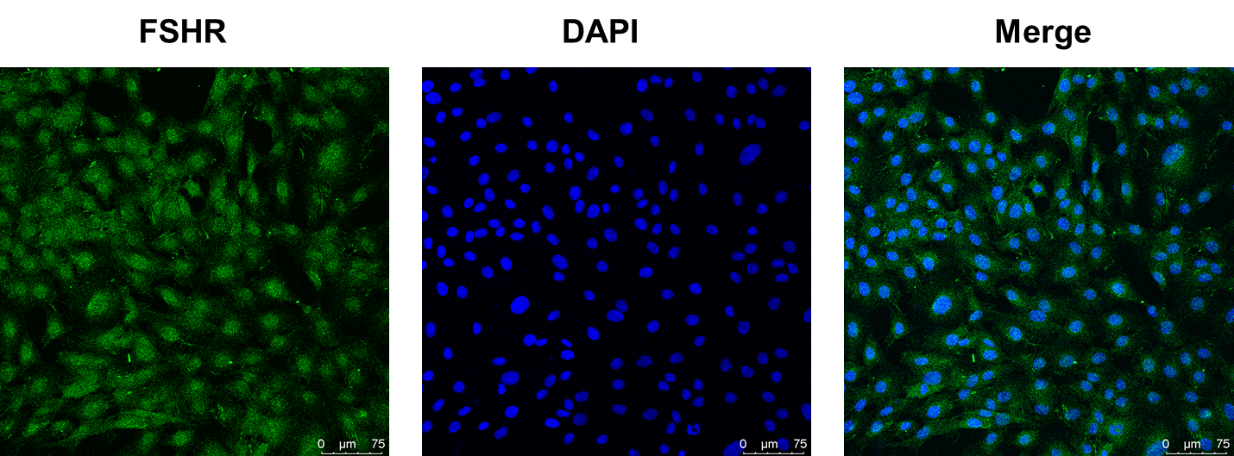 |
| --- |
| **Supplementary Figure 1.** FSHR expression in GCs was detected by immunofluorescence. FSHR positive cells were stained green. Nuclei were stained blue with DAPI. |

# Supplementary Table

# Supplementary Table 1. DEGs for three comparison groups

| Comparison groups | | Gene count | Genes involved |
| --- | --- | --- | --- |
| Control vs BHBA-1.2 m*M* | Up | 38 | *ENSBTAG00000053534, ENSBTAG00000054870, ENSBTAG00000051182, FOS, ACTN2, ENSBTAG00000051406, MSTRG.10889, GRIA3, ZNF234, ENSBTAG00000003937, LNX1, F2R, ENSBTAG00000053171, ENSBTAG00000051252, HIVEP3, ENSBTAG00000051086, SEMA5A, ENSBTAG00000055174, ENSBTAG00000051931, PLCD4, ENSBTAG00000049311, CYP7B1, GFRA3, CASC1, ENSBTAG00000050998, SYDE2, ENSBTAG00000049239, ENSBTAG00000052954, HNMT, CTTNBP2, RNF180, FOSB, PHOSPHO1, NXPE4, ENSBTAG00000048481, ESPNL, VEPH1, HAPLN4* |
|  | Down | 29 | *MSTRG.10367, LRP2BP, MCF2, ACTL6B, MX2, IFI44, TCAP, SYN2, AKAP5, AHRR, ENSBTAG00000015818, FRMD7, OAS1X, ISG15, ENSBTAG00000052874, ENSBTAG00000045588, AFMID, OAS1Y, S100A6, DKK1, GPNMB, G0s2, CDK5R1, IFI44L, ENSBTAG00000051767, MX1, ELMO3, PI15, LOXL4* |
| Control vs BHBA-1.2 m*M* | Up | 41 | *P2RY2, CNKSR2, ENSBTAG00000047365, SIX1, CBARP, ACTN2, HMGA1, MSTRG.8315, F2R, MED12L, ENSBTAG00000048358, ZNF283, NOTCH1, PTGDR, ZNF234, SLC16A3, JSP.1, PRDM12, SPIN4, SEMA5A, ENSBTAG00000054668, EEF1A1, ENSBTAG00000050998, SYNPO2L, CYP27B1, GMPR, EMILIN3, ACTC1, RASD1, NLRC5, NFASC, ENSBTAG00000054610, ICA1L, ENSBTAG00000050187, HNMT, SIM1, RNF180, MMP1, ALOX15, ENSBTAG00000048481, ENSBTAG00000051290* |
|  | Down | 40 | *ENSBTAG00000006872, IFI44, CYP17A1, AKAP5, PRRT1, FRMD7, SMPDL3A, DNAH10, ENSBTAG00000045523, ENSBTAG00000005315, MSTRG.5708, KCTD21, ENSBTAG00000045588, SFRP1, OAS1X, CDK5R1, EXTL1, IBSP, DACT3, PI15, ENSBTAG00000055021, ENSBTAG00000015251, ENSBTAG00000051119, ENSBTAG00000049728, G0s2, ENSBTAG00000044018, MSTRG.9210, ENSBTAG00000051767, FLVCR2, FAM166B, MSTRG.3690, ENSBTAG00000053347, CHL1, Pol, CTSO, INSYN2, NCKAP5, LOXL4, MXRA5, KIF21B* |
| BHBA-1.2 m*M*-BHBA-2.4 m*M* | Up | 19 | *EFCAB11, ENSBTAG00000037981, PIF1, ESR1, RAB3A, INKA1, PPP1R36, PRX, EEF1A1, NFASC, ENSBTAG00000052874, MSTRG.2692, CCDC157, ANGPT1, CLDN11, TMEM139, RNF43, ENSBTAG00000052520, MSTRG.10367* |
|  | Down | 40 | *ENSBTAG00000050331, ARC, RGS16, SLC38A8, SMPDL3A, FOSB, ENSBTAG00000051334, ENSBTAG00000049311, ENSBTAG00000048846, ENSBTAG00000005315, RFX3, CCDC96, CCDC141, ENSBTAG00000052805, HES4, ENSBTAG00000050534, KLF2, ENSBTAG00000050978, ENSBTAG00000050199, ENSBTAG00000049239, ENSBTAG00000052954, MSS51, GPR39, FOS, EXTL1, ENSBTAG00000055253, ENSBTAG00000054439, EGR1, ENSBTAG00000049338, JAKMIP3, GFRA3, ENSBTAG00000052394, ENSBTAG00000053111, ENSBTAG00000051086, ENSBTAG00000054870, ENSBTAG00000051406, ENSBTAG00000053347, Pol, ENSBTAG00000051119, CORO2B* |

# Supplementary Table 2. DEGs included in each profile

| **Profiles** | **Gene count** | **Genes involved** |
| --- | --- | --- |
| 0 | 3 | *PRRT1, LOC509118, CYP17A1* |
| 1 | 9 | *CDK5R1, FRMD7, AKAP5, DKK1, IFI44, LOC100298356, OAS1X, G0s2, S100A6* |
| 2 | 21 | *GPNMB, CCDC157, MCF2, LRP2BP, AFMID, ACTL6B, MX2, TCAP, ANGPT1, ISG15, LOC112441463, PRX, TMEM139, CLDN11, RNF43, SYN2, OAS1Y, AHRR, ENSBTAG00000052520,* *ENSBTAG00000052874, MSTRG.10367* |
| 3 | 17 | *IBSP, RFX3, RGS16,* *ENSBTAG00000005315, EXTL1, ENSBTAG00000006872, DNAH10, SMPDL3A, ARC, SFRP1, KCTD21, LOC786530, ENSBTAG00000050331, PARP, ENSBTAG00000050978,* *ENSBTAG00000051334, MSTRG.5708* |
| 4 | 4 | *EMILIN3, NFASC, MSTRG.2692, EEF1A1* |
| 5 | 15 | *CYP7B1, PLCD4, GFRA3, NXPE4, FOSB, HES4, CCDC141, SLC38A8, CCDC96, ENSBTAG00000048846, ENSBTAG00000049239, ENSBTAG00000049311, KLF2, ENSBTAG00000052805, ENSBTAG00000052954* |
| 6 | 15 | *PHOSPHO1, HAPLN4, CASC1, SYDE2, NLRC5, ESPNL, ALOX15, HNMT, SYNPO2L, CTTNBP2, VEPH1, RNF180, ENSBTAG00000048481, ENSBTAG00000050998, ENSBTAG00000051290* |
| 7 | 9 | *SIM1, ACTC1, ICA1L, GMPR, CYP27B1, RASD1, MMP1, ENSBTAG00000050187, ENSBTAG00000054610* |

,
